# UNDERSTANDING PRISM ACCEPTANCE THROUGH QUANTITATIVE AND QUALITATIVE MEASURES

**DOI:** 10.1093/geroni/igae098.0041

**Published:** 2024-12-31

**Authors:** Wendy Rogers, Tracy Mitzner, Sara Czaja, Walter Boot, Neil Charness

**Affiliations:** University of Illinois Urbana-Champaign, Champaign, Illinois, United States; Person in Design, Atlanta, Georgia, United States; Weill Cornell Medicine/Center on Aging and Behavioral Research, New York, New York, United States; Weill Cornell Medicine, New York, New York, United States; Florida State University, Tallahassee, Florida, United States

## Abstract

Long-term adoption of technology is influenced both by the design of the technology itself as well by the users’ attitudes towards the technology. Technology acceptance models have long indicated that individual differences in perceptions of ease of use and perceptions of usefulness are critical predictors of acceptance and use. Additional predictive factors include perceived enjoyment, facilitating conditions (e.g., instructional materials, social support), demographics (e.g., age, sex) and psychographics (e.g., personality, technology readiness). For PRISM 1.0 and PRISM 2.0 we implemented mixed methods to assess technology acceptance factors. We adapted standard survey measures of technology acceptance that were administered at baseline and after extended system use. We developed an interview that was administered at the end of the study to explore usage patterns, preferences, facilitators and barriers. Data from the technology acceptance assessments from PRISM 1.0 informed redesign decisions for the PRISM 2 system. We will present an overview of the quantitative and qualitative data to illustrate the value of the mixed methods approach and to provide insights about acceptance and adoption of novel technology system to older adults.

